# Multi-site, retrospective evaluation of an education campaign and order set update to support diagnostic stewardship for pneumococcal urinary antigen testing

**DOI:** 10.1017/ash.2026.10877

**Published:** 2026-10-06

**Authors:** David Alirezaei, Lauren C. Russell, Lee M. Amaya, Vy T. Nghiem, Corey M. Frederick, Lili Zhou, Timothy P. Gauthier

**Affiliations:** 1 Boca Raton Regional Hospital, USA; 2 Herbert Wertheim Cancer Institute, USA; 3 Baptist Health South Florida, USA

## Introduction

The pneumococcal urinary antigen test (PUAT) is a noninvasive assay that can be used to support the diagnosis of community-acquired pneumonia (CAP) due to *Streptococcus pneumoniae*. However, growing evidence has questioned its clinical utility due to low positivity rates and limited impact on antimicrobial management.^1–5^ Recent diagnostic stewardship evaluations suggest that PUAT rarely changes therapy despite substantial healthcare utilization and associated costs.

National guidelines advise against routine use of PUAT for all patients with CAP, suggesting it may be ordered in severe cases (a conditional recommendation with low quality of evidence).^6^ We aimed to evaluate a phased stewardship project consisting of provider education on appropriate ordering followed by removal of PUAT from electronic order sets.

**Figure 1.**
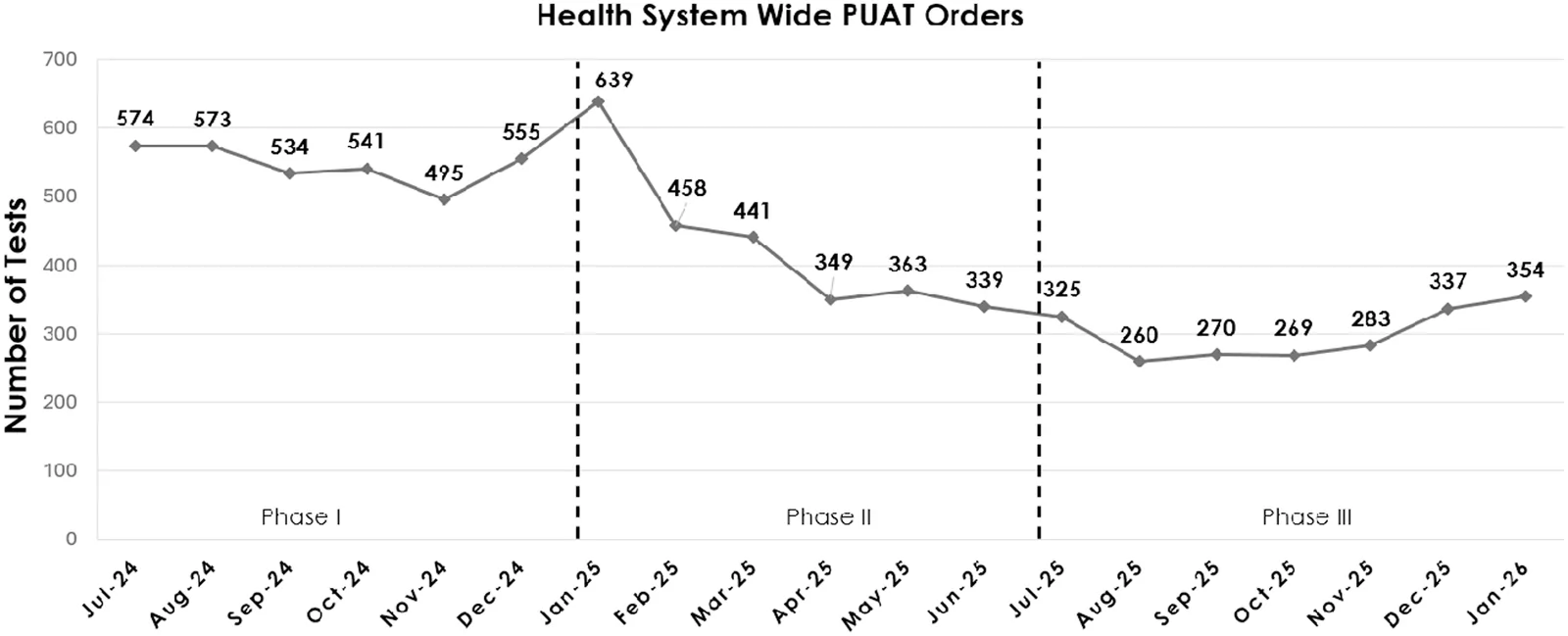
Monthly pneumococcal urinary antigen test (PUAT) utilization across the health system from July 2024 through November 2025. Provider education regarding appropriate PUAT utilization was implemented in phase II (January 2025–July 2025), followed by removal of PUAT from electronic order sets during phase III (July 2025). Progressive reductions in PUAT utilization were observed following diagnostic stewardship interventions.

## Methods

In this retrospective study, a diagnostic stewardship intervention consisting of provider education on appropriate PUAT utilization and subsequent removal from electronic order sets was implemented across nine academic community hospitals (30–950 beds). The study was conducted across three phases: preintervention (phase 1, July 2024–January 2025), education implementation (phase 2, January–July 2025), and order set PUAT removal (phase 3, July 2025–January 2026). The primary outcome was the rate of guideline-concordant PUAT ordering.

Secondary outcomes included PUAT positivity, impact of PUAT results on antibiotic therapy, days of atypical antibiotic (ie, antibiotics providing coverage for atypical pathogens) therapy avoided following PUAT-directed de-escalation, systemwide PUAT utilization, and associated laboratory costs. A cost of ＄28.34 was applied to each PUAT performed to estimate laboratory expenditures. To evaluate study outcomes, a system-generated report of all PUATs performed during the study period was obtained. Retrospective chart review was then performed on 100 patients per phase, with at least 20 positive PUAT results included per phase due to low overall positivity rates.

Data collected included demographics, antibiotic allergy history, immunocompromised status, CAP severity, microbiologic results, and antibiotic management. Patients were included if they were at least 18 years of age, had a first PUAT with a positive or negative result, and were hospitalized for management of CAP. Patients were excluded if they had hospital-acquired or ventilator-associated pneumonia, received no antibiotics, had a co-infection (eg, pneumonia with a non-pulmonary bloodstream infection), were pregnant or breastfeeding, or were incarcerated.

## Results

A total of 300 patients were included, with 100 patients in each phase. Baseline characteristics were generally similar across study phases. Median age was 77 years (IQR, 60–88) in phase 1, 75 years (IQR, 61–84) in phase 2, and 75 years (IQR, 64–83) in phase 3. Most patients identified as Hispanic (69%, 70%, and 67%, respectively), and severe CAP was present in 19%, 13%, and 23% of patients across phases. ICU admission within 48 hours of PUAT occurred in 13%, 8%, and 19% of patients, respectively.

Guideline-concordant PUAT ordering occurred in 18 patients in phase 1, 9 patients in phase 2, and 21 patients in phase 3 (*P* = .055). Following the targeted inclusion of positive PUAT results, positive tests were identified in 21, 21, and 20 patients in phase 1, 2, and 3, accordingly. Antibiotic therapy was narrowed within 24 hours following PUAT results in 7 patients in phase 1, 2 patients in phase 2, and 4 patients in phase 3 (*P* = .2). Among these 13 patients, 20 days of atypical antibiotic therapy were avoided.

Across the health system, mean monthly PUAT utilization was 509, 410, and 193 tests during phases I, II, and III, respectively. Estimated laboratory expenditures were approximately ＄101,000, ＄69,600, and ＄38,400 during phases 1, 2, and 3, respectively, corresponding to approximately ＄209,000 in total laboratory costs over the study period. Figure 1 displays total PUAT orders by month from phases I-III.

## Discussion

Pneumococcal urinary antigen testing demonstrated frequently negative results and infrequently altered antibiotic management, which is consistent with prior studies reporting low PUAT positivity rates and limited impact on antimicrobial prescribing.^1,2,5,7^ Appropriate ordering remained below 25% throughout the study period and was not significantly improved following either intervention. However, systemwide PUAT utilization decreased from approximately 509 tests per month during phase 1 to 193 tests per month during phase 3, suggesting that the interventions reduced overall testing volume, representing a diagnostic stewardship benefit, without improving guideline-concordant ordering. Therapy modification following positive PUAT results occurred infrequently, resulting in only 20 days of atypical antibiotic therapy avoided among 300 reviewed patients. While PUAT did not eliminate empiric atypical antibiotic exposure, it modestly reduced the duration of therapy in a small number of patients.

Similar findings have been reported by Falguera et al., who observed no clinical or economic benefit of PUAT-guided targeted therapy compared with empiric treatment, although antibiotics were narrowed in some patients.^3^ More recently, Davis et al evaluated 1,428 PUATs and found that only a single test resulted in a change in antimicrobial therapy, ultimately leading their institution to discontinue routine PUAT ordering.^4^ Our findings similarly demonstrated limited clinical impact despite substantial test utilization, further supporting consideration of PUAT removal as a diagnostic stewardship strategy.

Limitations include the low PUAT positivity rate, the potential for antibiotic decision-making to have been influenced by unmeasured clinical factors not captured in the data set, and seasonal variation in respiratory illness that may have influenced PUAT ordering across study phases. Additionally, this project was retrospectively designed, which relied on electronic health record documentation and was conducted within a single health system. Given PUAT’s low opportunity to meaningfully alter care pathways and minimal clinical impact when positive, these findings support reconsideration of routine PUAT availability and suggest its removal as an orderable test may be appropriate within diagnostic stewardship frameworks.

## Supporting information

10.1017/ash.2026.10877.sm001 Alirezaei et al. supplementary material 1 Alirezaei et al. supplementary material

10.1017/ash.2026.10877.sm002 Alirezaei et al. supplementary material 2 Alirezaei et al. supplementary material

## Supplementary material

The supplementary material for this article can be found at https://doi.org/10.1017/ash.2026.10877.
