# Supplementary material for: Multi-site, retrospective evaluation of an education campaign and order set update to support diagnostic stewardship for pneumococcal urinary antigen testing
Source: Antimicrob Steward Healthc Epidemiol. 2026 Oct 6;6(1):e308. doi: 10.1017/ash.2026.10877 (PMC13760396; doi:10.1017/ash.2026.10877)
Supplement: Supplementary file 1 [file S2732494X26108778sup001.docx]

Supplement 1: Educational 1-pager on pneumococcal and legionella urinary antigen test diagnostic stewardship
