# Supplementary material for: Multi-site, retrospective evaluation of an education campaign and order set update to support diagnostic stewardship for pneumococcal urinary antigen testing
Source: Antimicrob Steward Healthc Epidemiol. 2026 Oct 6;6(1):e308. doi: 10.1017/ash.2026.10877 (PMC13760396; doi:10.1017/ash.2026.10877)
Supplement: Supplementary file 2 [file S2732494X26108778sup002.docx]

Supplement 2: Baseline Characteristics for Cohorts with Chart Review

| **Patient Characteristic** | **Phase I**  **(n=100)** | **Phase II**  **(n=100)** | **Phase III**  **(n=100)** | **P-Value** |
| --- | --- | --- | --- | --- |
| Median age, years (IQR) | 77 (60-88) | 75 (61-84) | 75 (64-83) | 0.8 |
| Male gender, % | 48 | 53 | 37 | 0.7 |
| Race, %  White  Hispanic  African American  Asian  Middle Eastern  Unspecified | 25  69  3  2  0  1 | 23  70  3  1  1  2 | 21  67  7  0  0  5 | 0.4 |
| Antibiotic allergy, %  Penicillin  Cephalosporin  Sulfa | 22  2  6 | 15  2  5 | 15  2  5 | 0.3  0.1  0.9  0.8 |
| Immunocompromised, % | 10 | 4 | 10 | 0.2 |
| Severe CAP, % | 19 | 13 | 23 | 0.2 |
| ICU admission 48h of PUAT, % | 13 | 8 | 19 | 0.07 |

**Abbreviations**: IQR (interquartile range), CAP (community-acquired pneumonia), ICU (intensive care unit), PUAT (pneumococcal urinary antigen test)
